# Layer Dependence of Complex Refractive Index in CrSBr

**DOI:** 10.3390/ma17143430

**Published:** 2024-07-11

**Authors:** Chao Hu, Huanghuang Cheng, Jiayuan Zhou, Kai Zhang, Xue Liu, Yuxuan Jiang

**Affiliations:** 1 Material Science and Engineering, Anhui University, Hefei 230601, China; b21201072@stu.ahu.edu.cn (C.H.);; 2 School of Physics and Optoelectronics Engineering, Anhui University, Hefei 230601, China; 3 Center of Free Electron Laser and High Magnetic Field, Anhui University, Hefei 230601, China; 4 Institute of Physical Science and Information Technology, Anhui University, Hefei 230601, China

**Keywords:** two-dimensional antiferromagnet, complex refractive index, optical properties, layer dependence

## Abstract

CrSBr is a recently discovered two-dimensional anti-ferromagnet. It has attracted much attention due to its superior properties for potential optoelectronic and spintronic applications. However, its complex refractive index with layer dependence has not been systematically studied yet. In this work, we studied the room-temperature complex refractive indices of thin CrSBr flakes of different thicknesses in the visible light range. Using micro-reflectance spectroscopy, we measured the optical contrast of thin CrSBr flakes with respect to different substrates. The complex refractive index was extracted by modeling the optical contrast with the Fresnel equations. We extracted the band gap values of CrSBr in the few-layer limit. We determined the band gaps for monolayer, bilayer, and trilayer CrSBr to be 1.88 eV, 1.81 eV, and 1.77 eV, respectively. As a comparison, the band gap for multilayer CrSBr is outside our measured range, that is, below 1.55 eV. Our results suggest that the band gap of CrSBr decreases as thickness increases.

## 1. Introduction

The study of two-dimensional magnetic materials has been a topic of interest in the field of physics due to their potential applications in spintronics, topological superconductivity, and memory storage [1,2,3]. Among all van der Waals magnetic materials, CrSBr has attracted much attention due to its unique properties. As an A-type antiferromagnet [4], CrSBr has a high Néel temperature (up to 132 K) [5] and exhibits rich magnetic properties such as magnon-exciton interaction [6], negative magnetoresistance effect [7], triaxial magnetic anisotropy [8], and a strong magnetic proximity effect [9]. In addition, CrSBr possesses two remarkable features: air stability and semi-conductivity [10], making it a superior material for spintronic and device applications.

Currently, the optical properties of CrSBr are under intensive study. For example, Francisco Marques-Moros et al. measured its temperature- and magnetic-field-dependent photoluminescence (PL) at different thicknesses [11]. Amit Pawbake et al. measured the low-temperature magneto–optical properties of bulk CrSBr under hydrostatic pressure [12]. Linhart, W. M. et. al. found that its optical properties are strongly affected by its magnetic states in the bulk [13]. However, the complex refractive index of CrSBr has not been studied, particularly its layer dependence. As it is known, physical properties heavily rely on layer thickness [14,15,16], and it is expected that CrSBr exhibits similar properties. For example, quantum confinement could induce a layer-dependent electronic band gap, which affects its absorption and photoluminescence [11,17]. Furthermore, the competition between interlayer exchange, magnetic anisotropy, and Zeeman energy gives rise to a layer-dependent spin reorientation effect [4]. Due to the close connection between the optical, electronic, and magnetic properties of CrSBr, studying the layer dependence of the refractive index could facilitate the understanding of these important physical properties. Moreover, This information could provide important references for their application in photonic and optoelectronic devices.

There are several methods for determining the refractive indices of thin film materials in experiments, which include the Kramers–Kronig analysis [18], the elliptic polarization method [19], and so on. For microscale samples, the micro-reflectometry method is particularly powerful and well suited for the characterization of layered, two-dimensional materials [20,21,22]. In this paper, we use the micro-reflectometry method and Fresnel equation to extract the complex refractive index of mechanically exfoliated single, double, triple, and multi layer CrSBr in the visible light range. We identify their band gap size and find a consistent redshift as layer thickness increases. 

## 2. Materials and Methods

### 2.1. Sample Preparation

**Figure 1:**
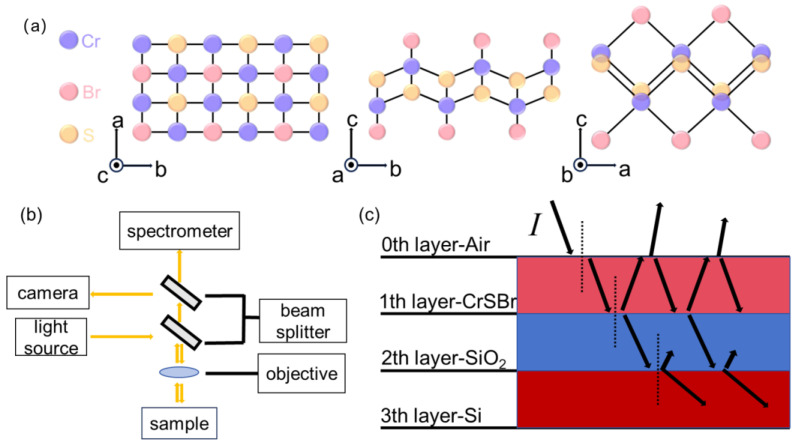
(**a**) Atomic structures of CrSBr viewed from different axes. (**b**) Schematic drawing of the home-built optical measurement setup. (**c**) Schematic drawing of the multi-layer thin-film optical model.

The CrSBr single crystals were synthesized using a chemical vapor transport method [4]. The crystal structure of CrSBr is shown in Figure 1a. In the a-b plane, a chromium sulfide double layer is sandwiched by Br atoms, and the layers are stacked along the c-axis. The space group of CrSBr is Pmmn, and the lattice constants of a unit cell are a = 3.50 Å, b = 4.76 Å, and c = 7.96 Å.

For sample preparations, we used the polydimethylsiloxane (PDMS)-assisted method to transfer thin flakes onto silicon wafers. First, we mechanically exfoliated single-crystal CrSBr into thin flakes with adhesive tapes and stamped them onto the PDMS. We then quantified the layer number of these flakes by color channel analysis and Raman spectroscopy. Such a method has been shown to be an accurate and fast way to determine thickness for 2D materials [22,23]. After locating the CrSBr flakes with the desired thickness, the PDMS was flipped over, aligned, and stamped onto the target substrate using a transfer stage. To detach the thin flakes from the PDMS, the substrate was heated up to 50 Celsius for 5 min before the PDMS was lifted. After the transfer process, we also performed Raman spectroscopy (Horiba Odyssey, Lyon, France) of our few-layer samples with an excitation laser of 633 nm to further confirm their thickness. In total, we have prepared samples with single- (1L), double- (2L), triple- (3L), and multi-layer (ML) thin flakes. Knowing the layer number and also the 1L thickness [24], we can determine the thickness of 1L, 2L, and 3L CrSBr flakes to be 0.8, 1.6, and 2.4 nm, respectively.

### 2.2. Optical Contrast Measurement

In this work, we followed the methods in refs [25,26] to extract complex refractive indices from the optical contrast measurements. Here, the optical contrast is defined as that in refs [20,21,22,26]:

(1) $$C=\frac{I_{2D}-I_{s}}{I_{2D}+I_{s}},$$

where $I_{2D}$ and $I_{s}$ are the reflectance from a CrSBr/SiO_2_/Si sample and a SiO_2_/Si substrate with the same SiO_2_ thickness, respectively. Since refractive indices ($\tilde{n}=n-ik$) are complex numbers, it requires multiple measurements to determine their values. In this work, we chose three different measurements with 90, 250, and 310 nm SiO_2_ thicknesses for 1–3L samples. The reason is that (a) these thicknesses are commercially readily available, and (b) these thicknesses are relatively well separated, and the contrast dips have very little overlap, favorable for the determination of the refractive index. We note that the choice of the SiO_2_ thicknesses is not unique. For the 1L, 2L, and 3L CrSBr flakes, they were transferred onto Si substrate. For ML samples, we fixed the SiO_2_ thickness to be 310 nm and varied the thin flakes thickness to be 11 nm, 14 nm, and 19 nm. The choice of the ML flake thickness is rather arbitrary, provided that the thickness is neither too thin, so that the refractive indices remain unchanged, nor too thick for light penetration.

The optical contrast was measured with a home-built setup, and the schematic drawing of the setup is shown in Figure 1b. It consists of a metallurgical microscope (SOPTOP, CX40M, Ningbo, China), a modified trinocular port, an external tungsten halogen light source, and a fiber-coupled spectrometer (Ocean Optics USB2000+ type, Orlando, FL, USA). The incident light from a tungsten halogen lamp was reflected from a 50:50 beam splitter. It was then focused onto samples through a 50×objective (SOPTOP, NA = 0.55) with a beam spot of 5 μm. The reflected lights passed through the beam splitter again and were collected with a 400 um multimode fiber. The fiber delivered the signal light into the spectrometer for spectra analysis. In addition, we inserted a removable 50:50 beam splitter above the first beam splitter for imaging.

To measure the reflection contrast, we first recorded a spectrum $I_{2D}$ for lights reflected from samples. We then moved to a nearby spot without a sample to take a reference spectrum $I_{s}$. We calculated optical contrasts easily following Equation (1). During the measurements, we kept the incident light intensity the same to avoid any artifacts. All measurements were performed at room temperature and ambient pressure.

### 2.3. Extraction of the Refractive Index

In order to extract the refractive index, we need to consider interference effects between layers, which is schematically shown in Figure 1c. We denote the air, sample, SiO_2_, and Si as 0th, 1st, 2nd, and 3rd layers, respectively. For perpendicular incident light, the reflected light can be calculated using Fresnel equations. For bare substrates, $I_{s}$ can be calculated as

(2) $$I_{s}=I_{0}{\left|\frac{r_{02}+r_{23}e^{-2i\Phi_{2}}}{1+r_{02}r_{23}e^{-2i\Phi_{2}}}\right|}^{2},$$

where $r_{ij}={(\tilde{n}}_{i}-{\tilde{n}}_{j}$)$/({\tilde{n}}_{i}+{\tilde{n}}_{j})$, ${\tilde{n}}_{i}$ is the complex refractive index of the ith layer, $\phi_{i}=2\pi n_{i}d_{i}/\lambda$, and λ are the wavelength of the incident light. For samples, the reflected light intensity $I_{2D}$ is modified to

(3) $$I_{2D}=I_{0}\left|\frac{r_{01}e^{i(\Phi_{1}+\Phi_{2})}+r_{12}e^{-i(\Phi_{1}-\Phi_{2})}+r_{23}e^{-i(\Phi_{1}+\Phi_{2})}+r_{01}r_{12}r_{23}e^{i(\Phi_{1}-\Phi_{2})}}{e^{i(\Phi_{1}+\Phi_{2})}+r_{01}r_{12}e^{-i(\Phi_{1}-\Phi_{2})}+r_{01}r_{23}e^{-i(\Phi_{1}+\Phi_{2})}+r_{12}r_{23}e^{i(\Phi_{1}-\Phi_{2})}}\right|.$$

By fitting the experimental data to Equations (1)–(3), the complex refractive index of CrSBr flakes can be determined. More specifically, we create an array of n and k with equally spaced N values within a certain value range. This allows us to generate an N × N matrix to represent different combinations of n and k (i.e., complex refractive index). For a given CrSBr layer (1L–3L) and a given wavelength, we have a set of data for different SiO_2_ thicknesses. Our task is to find the matrix element that minimizes the total errors of the data set between the theoretical and experimental optical contrast. Then, this matrix element gives the n and k of the complex refractive index for the wavelength of the sample. This procedure is repeated throughout the measured wavelength range. For the case of ML, the procedure is similar except that the varying thickness is CrSBr instead of SiO_2_.

## 3. Results and Discussion

**Figure 2:**
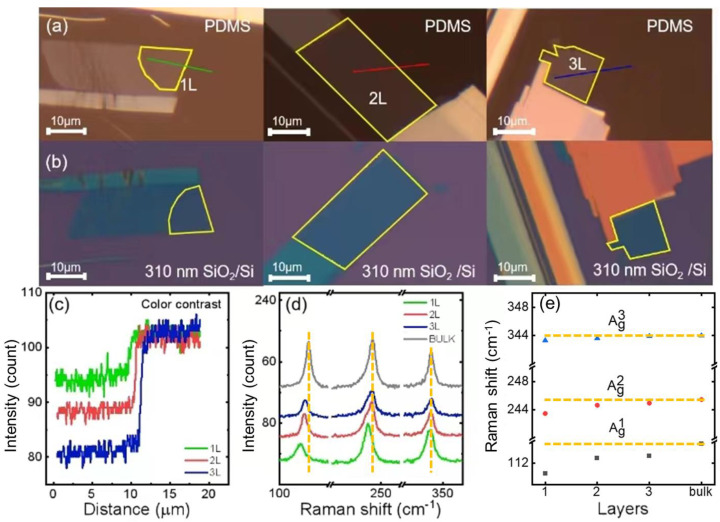
Sample characterizations. (**a**) Optical images of mechanically exfoliated few-layer CrSBr flakes on PDMS. The yellow lines in each panel show the regions of the 1L, 2L, and 3L CrSBr flake, respectively. (**b**) Optical images of CrSBr flakes transferred onto SiO_2_/Si substrates. The thickness of SiO_2_ is shown. (**c**) The color contrast intensity of the 1L, 2L, and 3L CrSBr flake along the solid-colored lines indicated in panel (**a**). (**d**) Raman spectra measured on the 1L, 2L, 3L, and bulk CrSBr flakes. (**e**) The layer dependence of the extracted Raman peak positions of the three modes in panel (**d**).

Figure 2a shows the optical images of 1L, 2L, and 3L CrSBr flakes on PDMS, and Figure 2b shows their optical images after transferring onto 310 nm SiO_2_/Si substrates. Their thicknesses are quantified by color channel intensity analysis [23]. Figure 2c shows the color intensity profile of the CrSBr flakes on the PDMS along the solid lines drawn in Figure 2a. For all three samples, the intensity for PDMS has a maximum value of ~103, and it reduces to ~95, ~88, and ~80 for 1L, 2L, and 3L, respectively. The intensity roughly decreases at a step size of ~8 units as the layer number increases. In other words, each layer of CrSBr induces eight units of intensity change. Therefore, by comparing the intensity difference between the regions with and without a sample, we can confirm the thickness of our samples.

We also confirmed the thickness by Raman analysis after transferring the samples onto the SiO_2_/Si substrates. Figure 2d shows the layer-dependent Raman spectra of our prepared CrSBr flakes. We observed the A_1g_, A_2g_, and A_3g_ peaks at the energy positions of 113 cm^−1^, 245 cm^−1^, and 345 cm^−1^, respectively. Figure 2e summarizes these Raman peak positions as a function of the thickness. The positions of A_1g_ and A_2g_ show blueshifts as the layer number increases, while that of A_3g_ is layer-independent. Our Raman result is consistent with the reported literature for few-layer CrSBr flakes [27].

**Figure 3:**
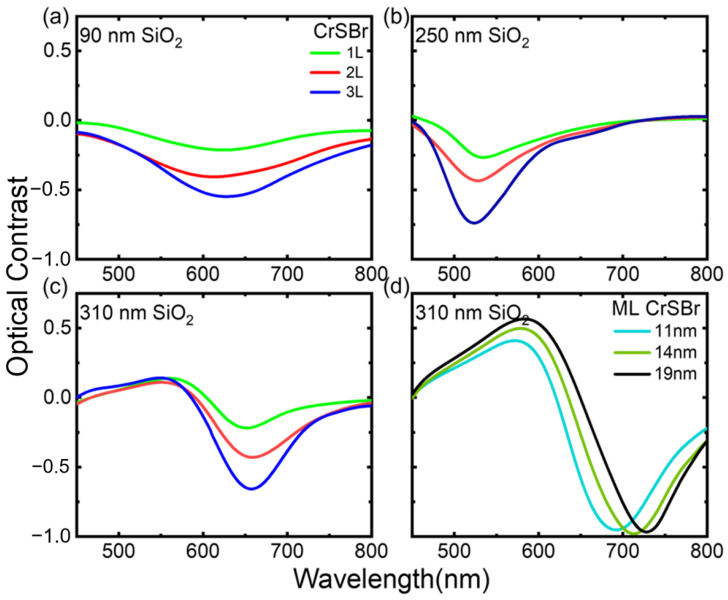
Optical contrast measurements. (**a**–**c**) Optical contrast of 1L, 2L, and 3L CrSBr thin flakes on SiO_2_/Si substrates with SiO_2_ thicknesses of (**a**) 90 nm, (**b**) 250 nm, and (**c**) 310 nm in the wavelength range 450–800 nm. (**d**) Optical contrast of 11 nm, 14 nm, and 19 nm CrSBr ML thin flakes on SiO_2_/Si substrates with SiO_2_ thicknesses of 310 nm.

Figure 3a–c show the measured optical contrast of 1L, 2L, and 3L samples on the SiO_2_/Si substrates with SiO_2_ thicknesses of 90 nm, 250 nm, and 310 nm. Figure 3d shows the measured optical contrast of the 11 nm, 14 nm, and 19 nm of ML CrSBr flakes on the 310 nm SiO_2_/Si substrate. We observed strong dips in all samples, but the dip positions vary with the silicon oxide thickness. The samples on 250 nm SiO_2_ have dips slightly above 500 nm, while the samples on 90 and 310 nm have dips at around 650 nm. The location of the reflection contrast dip depends primarily on the thickness of the sample and SiO_2_, due to the interference between the reflected light within each layer [28,29]. This is mostly evidenced in the ML samples, where the SiO_2_ thickness is fixed and the sample thickness varies. As the thicknesses increase, the optical path becomes longer, and hence, the optical contrast dip redshifts. Similar phenomena are also observed in refs. [26,30,31]. In addition, as the layer number increases, the optical contrast varies more prominently. This is expected as more reflections/absorptions occur with increasing thicknesses.

**Figure 4:**
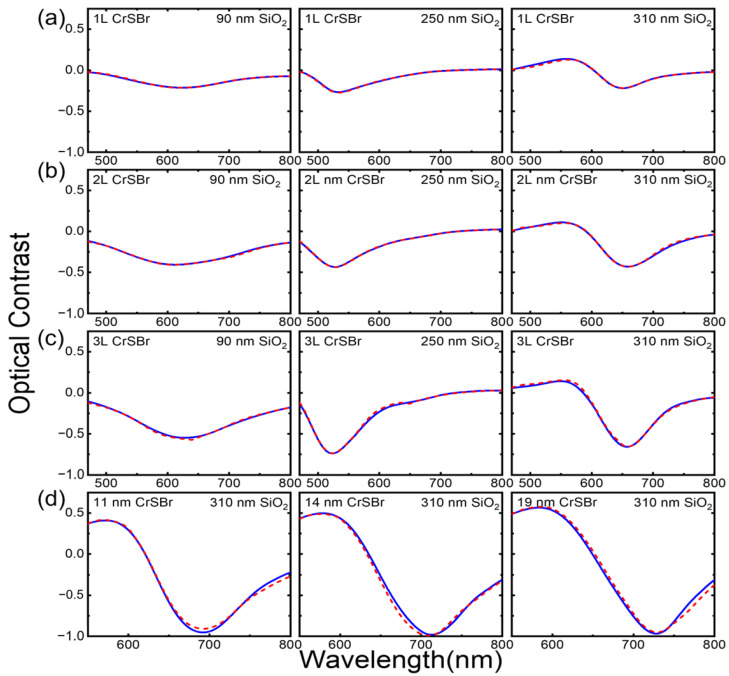
Comparison between the experimental and modeled optical contrast on (**a**) 1L, (**b**) 2L, (**c**) 3L, and (**d**) ML CrSBr flakes. The blue lines are experimental results while the red dotted lines are modeling results. In (**a**–**c**), the panels from left to right in each row correspond to the optical contrast of the CrSBr flakes on SiO_2_/Si substrates with SiO_2_ thicknesses of 90 nm, 250 nm, and 310 nm, respectively. In (**d**), the panels from left to right correspond to the optical contrasts of 11 nm, 14 nm, and 19 nm CrSBr on 310 nm SiO_2_/Si substrates, respectively.

Figure 4 shows the comparison between the experimental data and the fitting results using the procedures in Section 2.3. The relevant parameters used here include the refractive indices of air [32], SiO_2_ [33], and Si [34]. The thicknesses of the SiO_2_ layer used here are 90, 250, and 310 nm. The thicknesses of the 1L, 2L, and 3L CrSBr are 0.8, 1.6, and 2.4 nm, respectively, and those used for the ML are 11, 14, and 19 nm. The red lines are the theoretical fitting results, and the blue lines are the experimental data. As is shown in Figure 4, the fitting reasonably agrees with the experiment data.

**Figure 5:**
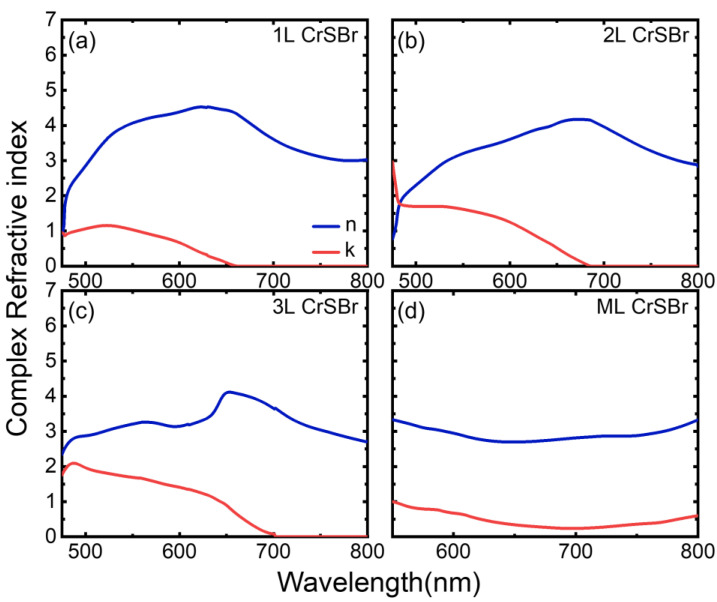
Extracted complex refractive index for (**a**) 1L, (**b**) 2L, (**c**) 3L and (**d**) ML of CrSBr flakes. The real parts (n) are indicated by the blue lines while the imaginary parts (k) are indicated by the red lines.

Figure 5 summarizes the extracted complex refractive indices for the 1L, 2L, 3L, and ML CrSBr within the measured wavelengths. The real and imaginary parts of the complex refractive index are represented by the blue and red lines in the figure, respectively. As is known, the real part describes the refraction, and the imaginary part represents the absorption of the materials. While the refractions are qualitatively similar between all samples, the absorptions show distinct behaviors among them. To explain the particular line-shape of the refractive index, it requires first-principle calculation to fully understand its electronic band structure [35]. However, the band gap information is readily available. A typical signature of band gap absorption is a sudden turn-on of absorption in spectra as electrons only absorb photons above band gap energy [13]. In Figure 5, with decreasing wavelengths, the absorptions for the 1L to 3L remain at zero (flat lines) and then abruptly start to increase below certain wavelengths. We can therefore extract the band gap of 1L, 2L, and 3L CrSBr to be around 1.88 eV (659 nm), 1.81 eV (685 nm), and 1.77 eV (700 nm), respectively. The red shift of the band gap is likely due to the quantum confinement effect in the vertical direction [11]. Such an effect is commonly seen in 2D materials in the few-layer limit [36,37,38]. On the contrary, the band gap behavior is not identified in the absorption of the ML sample. Hence, the band gap for the ML sample is beyond our measurement range, suggesting a band gap smaller than 1.55 eV (800 nm). Consequently, we can also deduce the excitonic binding energy for the few-layer samples. From photoluminescence measurements [11], the exciton energy is about 1.2 eV. Therefore, the excitonic binding energies are roughly about 0.6 eV for few-layer samples. As a comparison, the exciton binding energies for single-layer MoS_2_, WS_2_, MoSe_2_, and WSe_2_ are ~0.54 eV [39], 0.71 eV [40], 0.55 eV [41], and 0.37 eV [42], respectively.

Before closing, we note that it would be tempting to extract the magnetic properties from the optical properties in CrSBr as discussed in the introduction. However, this is difficult in our study as the system does not exhibit any magnetic order state at the measurement temperatures. It would be interesting in the future to study the temperature dependence of the complex refractive index and reveal the information about its relationship with magnetic order states.

## 4. Conclusions

We measured the room-temperature optical contrasts of exfoliated 1L, 2L, and 3L CrSBr thin flakes on SiO_2_/Si substrates from 450 nm to 800 nm. By fitting the measured optical contrasts with Fresnel equations, we studied the layer dependence of the complex refractive index in CrSBr. We identify a red shift in the band gap value with increasing layer thickness. More specifically, the band gap sizes of 1L, 2L, and 3L CrSBr are estimated to be 1.88 eV, 1.81 eV, and 1.77 eV, respectively. However, no signature of band gap absorption is observed in the ML sample within our measured range. This indicates that the band gap for the bulk sample is below 800 nm. In addition, we also deduce the excitonic binding energy to be 0.6 eV for the few-layer samples. Our results provide new information about the layer dependence of the optical properties and electronic structures of CrSBr, which could be useful for their future optoelectronics applications and device designs.

## Data Availability

The raw data supporting the conclusions of this article will be made available by the authors on request.
